# MicroRNA-597 Suppresses Gastric Cancer Invasion and Progression via *RUNX1* Targeting, an Effect Attenuated by the Long Non-Coding RNA *KCNQ1OT1*

**DOI:** 10.3390/ijms27125368

**Published:** 2026-06-14

**Authors:** Alejandra Sandoval-Borquez, Wilda Olivares, Francisco J. Carvajal, Pablo M. Santoro, Carolina Bizama, Yáreni Ávalos-Guajardo, Keila Torres, Marcelo Garrido, Enrique Norero, Andrew F. G. Quest, Alejandro H. Corvalan

**Affiliations:** 1 Advanced Center for Chronic Diseases (ACCDiS), Faculty of Medicine, Pontificia Universidad Católica de Chile, Santiago 8330024, Chile; alejandra.sandoval@pucv.cl (A.S.-B.); wdolivar@uc.cl (W.O.); fvjcarvajal@gmail.com (F.J.C.); p.santoro.v@gmail.com (P.M.S.); cbizamas@uc.cl (C.B.); yareni.delourdes@gmail.com (Y.Á.-G.); keilatorresizarra@gmail.com (K.T.); 2 Medical Technology School, Faculty of Sciences, Pontificia Universidad Católica de Valparaíso, Valparaiso 2370363, Chile; 3 Department of Hematology and Oncology, Faculty of Medicine, Pontificia Universidad Católica de Chile, Santiago 8330032, Chile; drmgarrido@gmail.com; 4 Department of Pathology, Faculty of Medicine, Pontificia Universidad Católica de Chile, Santiago 8320000, Chile; 5 Esophagogastric Surgery Unit, Department of Digestive Surgery, Hospital Dr. Sotero del Rio, Santiago 8207257, Chile; drenriquenorero@gmail.com; 6 Cellular Communication Laboratory, Faculty of Medicine, University of Chile, Santiago 8380453, Chile; aquest@u.uchile.cl; 7 Center for Studies on Exercise, Metabolism and Cancer (CEMC), Faculty of Medicine, University of Chile, Santiago 8380453, Chile; 8 Nucleus of Biology and Genetics, Institute of Biomedical Sciences (ICBM), Faculty of Medicine, University of Chile, Santiago 8389100, Chile; 9 Advanced Center for Chronic Diseases (ACCDiS), Faculty of Medicine, University of Chile, Santiago 8389100, Chile

**Keywords:** gastric cancer, *KCNQ1OT1*, microRNA-597, *RUNX1*, patient-derived organoids

## Abstract

Aberrant expression of multiple microRNAs has been reported in gastric cancer. In particular, microRNA-597 has been associated with poor survival rates but is not yet well characterized. Seventy-five clinical samples, four cell lines, and two patient-derived organoids were evaluated for the expression of microRNA-597 and its target genes. microRNA-597 was transiently transfected for analysis of cell migration, invasion, wound healing, colony formation, and cell viability, and its regulation by long non-coding RNAs was explored using the TCGA-STAD and LncBook tools. In clinical samples, low expression of microRNA-597 was associated with the intestinal subtype (*p* = 0.002) and stages III and IV (*p* = 0.048). All functional readouts were reduced after microRNA-597 transfection, including colony formation, in patient-derived organoids. Among target genes, *RUNX1* was directly regulated by microRNA-597. Other cell invasion genes were dependent on *RUNX1* as a hub for regulation. Analysis of the Intersection between long non-coding RNAs co-expressed with *RUNX1* and those with the highest microRNA-597 prediction binding identified *KCNQ1OT1* as the top transcript. Silencing of *KCNQ1OT1* and co-expression in clinical samples suggest the existence of a *KCNQ1OT1*/microRNA-597/*RUNX1* network. The results indicate that microRNA-597 directly suppresses *RUNX1*, while KCNQ1OT1 modulates this interaction. Our approach enabled the simultaneous analysis of dysregulation in three families of transcripts in gastric cancer progression.

## 1. Introduction

Despite a global decline in mortality, gastric cancer (GC) is not only the fifth most common malignancy but also the third leading cause of cancer-related deaths worldwide [1]. The incidence of GC shows considerable geographic variation. The regions of East Asia, Eastern Europe, and Latin America have the highest prevalence, while Africa and Western Asia show low overall prevalence [2]. This geographical heterogeneity highlights the importance of developing a more precise understanding of the coding and non-coding genomic diversity in GC among highly impacted populations. A unique landscape of GC has been revealed by studies focusing on East Asia. This includes mutations in coding genes related to proteins involved in the immune evasion system, coding genes associated with cell adhesion (i.e., extracellular domains of the *CDH1* gene), and the activation of transcripts that drive neoplastic progression (i.e., *PIGR* and *SOX9*) [3]. In Eastern European populations, a unique divergence in mutational patterns has been reported, highlighting the predominance of the G:C > A:T transition in non-coding regions [4]. Latin America presents a novel opportunity to analyze the genomic diversity of GC. The elevated frequency of Epstein–Barr virus (EBV) infection and the low frequency of human epidermal growth factor receptor 2 (HER2) and programmed death-ligand 1 (PD-L1) are unique features of tumors found in this region [5].

Non-coding genes represent 98% of the human genome, with nearly 80% expressed as housekeeping genes and transcripts derived from complementary sequences of coding genes [6]. Based on the size of their sequence, these transcripts can be divided into two families: small (~20–200 nucleotides (nt)) and long non-coding (lnc) RNAs (200 nt to ~100,000 nt [7]). MicroRNAs (miRs) represent the most important member of the small RNA family and induce endonucleolytic degradation at the untranscribed 3′ region, regulating the expression of more than 30% of all mRNAs [8]. miRs impact multiple cellular functions by targeting master regulatory genes or “hub genes” [9]. lncRNAs exhibit a greater diversity in function, including the ability to sponge miRs and thereby attenuate their endonucleolytic degradation [6]. Therefore, mRNAs and lncRNAs have to compete for the same pool of miRs. Aberrant expression signatures of multiple miRs have been reported in human tumors [10]. This aberrant expression can be traced to specific miRs as a reflection of the developmental lineage and differentiation state of tumors [11]. In GC, Woo et al. [12] identified 14 candidates for prognosis of survival. These candidates include a group of uncharacterized or poorly characterized miRs in GC [13,14,15,16,17]. We chose to focus in an unbiased manner on miR-597 because it has been described as the most relevant miRNA in multiple tumors, particularly in those located in the gastrointestinal (GI) tract [18,19,20,21]. Although it has been established that miRs target multiple hub genes in GC [22], the master regulatory gene Runt-related transcription factor 1 (*RUNX1*) has not yet been studied in this field. *RUNX1* belongs to the RUNX family, which comprises three evolutionarily conserved genes (*RUNX1*, *RUNX2*, and *RUNX3*) [23]. While initially cataloged as a tumor suppressor gene in hematological malignancies, *RUNX1* is emerging as an oncogene in gastrointestinal and other solid tumors [24]. In this study, we show that loss of function of miR-597 is critical in promoting invasion and metastasis in GC through *RUNX1*, which is a hub for the expression of cell invasion and tumor progression-related genes. Moreover, we suggest that miR-597 is sponged by the lncRNA *KCNQ1OT1*, thereby attenuating *RUNX1* endonucleolytic degradation.

## 2. Results

### 2.1. Clinicopathological Associations with miR-597

We assessed the clinical significance of miR-597 in 75 GC patient samples by determining the mean expression (2^−ΔCT^ method) [25]. Among clinicopathological variables, the intestinal subtype and tumors at stages III and IV were associated with low expression of miR-597 (Table 1).

### 2.2. High miR-597 Expression Suppresses Gastric Cancer Cell Functions

**Figure 1:**
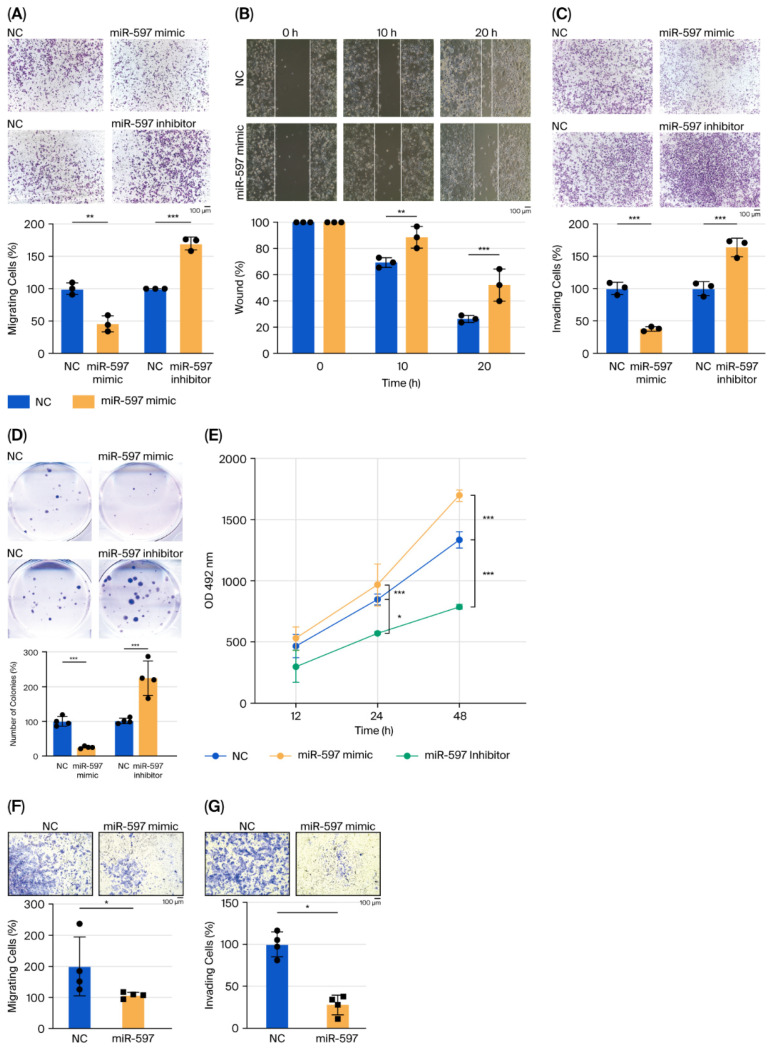
Functional assays of miR-597 in gastric cancer cell lines. (**A**) Cell Migration assay was performed in miR-597 mimic- and inhibitor-transfected AGS cells (*N* = 3). Representative images of the number of migrating cells (upper panel) and their quantification (lower panel) after 18 h are shown. Migrating cells were expressed as a percentage relative to the corresponding NC condition, which was set as 100% for each experiment. (**B**) Wound healing assay was performed in miR-597 mimic-transfected AGS cells (*N* = 3). Representative images of the kinetics of migration ability evaluated at 3 time intervals (upper panel) and quantified (lower panel) by the percentage of cells per replicate. For each experiment, the wound area at 0 h was set as 100%, and the remaining wound area at 10 and 20 h was determined relative to its corresponding 0 h value. (**C**) Cell Invasion assay was performed in miR-597 mimic-transfected AGS cells (*N* = 3). Cells were seeded in serum-free media onto the top filter (upper chamber) coated with Matrigel, and the lower chamber contained the chemoattractant (10% FBS media). Invading cells were counted in 5 randomly selected visual fields under the microscope, and their invasive ability was quantified and expressed as a percentage relative to the NC condition, which was set as 100% for each experiment. Representative images of the number of invading cells (upper panel) and their quantification (lower panel) are shown. (**D**) Colony formation assay was performed in miR-597 mimic- and inhibitor-transfected AGS cells (*N* = 4). After 14 days, surviving colonies were stained, scanned at high resolution (≥50 cells per colony), and counted. Colony formation was expressed as a percentage relative to the corresponding NC condition, which was set as 100% for each experiment. Representative images of the number of colonies (upper panel) and their quantification (lower panel) are shown. (**E**) MTS Cell viability assay was performed in miR-597 mimic- and inhibitor-transfected AGS cells (*N* = 3). Viability was evaluated at 3 time intervals with absorbance at 492 nm. (**F**) Cell Migration assay was performed in miR-597 mimic- and inhibitor-transfected MKN74 cells (*N* = 4). Representative images of the number of migrating cells (upper panel) and their quantification as a percentage (lower panel) after transfection. (**G**) Cell Invasion assay was performed in miR-597 mimic-transfected MKN74 cells (*N* = 4) as described in (**C**). Representative images of the number of invading cells (upper panel) and their quantification as a percentage (lower panel) after transfection are shown (* *p* < 0.05; ** *p* < 0.01 and *** *p* < 0.001). Data are presented as mean ± SD.

To explore the role of miR-597 as a tumor suppressor gene, we examined its endogenous expression in four different GC cell lines using quantitative RT-PCR normalized to RNU6B. The analysis showed a similar expression of miR-597 in all cell lines (Figure S1A). To investigate the functional role of miR-597, we performed gain- and loss-of-function experiments in AGS cells. The transfection efficiency is shown in Figure S1B. The gain of function of miR-597 resulted in significantly reduced cell migration in transwell assays compared to negative controls, while inhibition of miR-597 enhanced cell migration (Figure 1A). Wound healing assays further demonstrated that the miR-597 mimic led to decreased wound closure when evaluated at 10 and 20 h posttransfection, indicative of impaired migration of these cells (Figure 1B). In invasion assays using Matrigel-coated transwells, AGS cells transfected with the miR-597 mimic exhibited reduced invasiveness 20 h posttransfection compared to negative controls. Conversely, the inhibition of miR-597 increased cell invasion (Figure 1C). Clonogenic assays after transfection with the miR-597 mimic resulted in a significant decrease in colony formation over 14 days, whereas inhibition led to an increase in the number of colonies (Figure 1D). These results suggest that miR-597 suppresses both migratory and invasive cell behavior, and modulates the clonogenic potential of GC cells. The impact of miR-597 expression on cell viability was evaluated in MTS assays. To this end, AGS cells were transfected with the miR-597 mimic or inhibitor, and absorbance at 492 nm 12 h, 24 h, and 48 h posttransfection was evaluated. In miR-597 mimic-transfected cells, cell viability was reduced compared to controls, whereas miR-597 inhibitor-transfected cells showed increased viability (Figure 1E). The Results obtained with the Trypan blue assays further confirmed the MTS findings (Figure S1C). To validate the tumor-suppressive effects of miR-597, we transfected a second GC cell line, MKN74, with the miR-597 mimic. The transfection efficiency is shown in Figure S1B. Gain-of-function experiments resulted in a significant reduction in MKN74 migration compared to the negative controls (Figure 1F). Similarly, invasion assays revealed a decreased invasive capacity in miR-597 mimic-transfected MKN74 cells (Figure 1G). Collectively, these data support the notion that miR-597 functions as a tumor suppressor gene in GC cells.

### 2.3. miR-597 Inhibits Tumorigenic Behaviors in Gastric Cancer Patient-Derived Organoids

**Figure 2:**
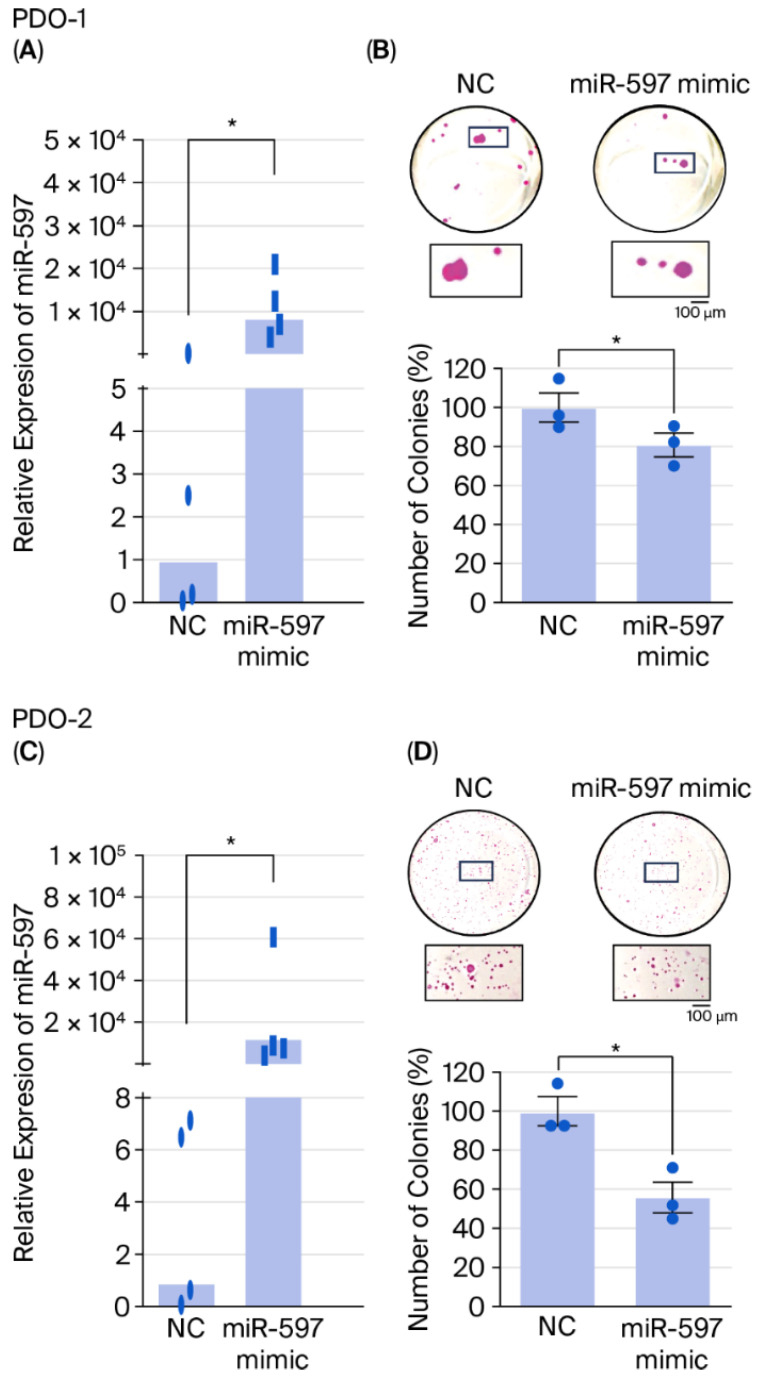
Functional assays of miR-597 in two patient-derived organoids (PDOs). (**A**,**C**) Expression levels of miR-597 in PDO-1 and -2, respectively, after transfection with the miR-597 mimic (*N* = 4). (**B**,**D**) Colony formation assay after transfection with the miR-597 mimic for 14–21 days was performed (*N* = 3). Surviving colonies were stained and scanned for counting (≥50 cells per colony). Representative images of the number of colony-forming cells (**B**,**D**, upper panels) and their quantification (**B**,**D**, lower panels) after transfection are shown (* *p* < 0.05). Data are presented as mean ± SEM.

We extended our studies to patient-derived organoids (PDOs) to assess the relevance of miR-597 in a more physiologically relevant model. Significantly higher expression levels of miR-597 were observed after transfection with the miR-597 mimic in both organoids (Figure 2A,C). This finding confirms the efficiency of transfection in PDOs. Next, a significant reduction in organoid colony formation was observed after transfection with the miR-597 mimic compared to negative controls (Figure 2B,D). These findings suggest that miR-597 prevents tumor growth in PDOs, underscoring its potential role as a tumor suppressor gene in GC.

### 2.4. miR-597 Reduces the Expression of Genes Associated with Cell Invasion and Tumor Progression

**Figure 3:**
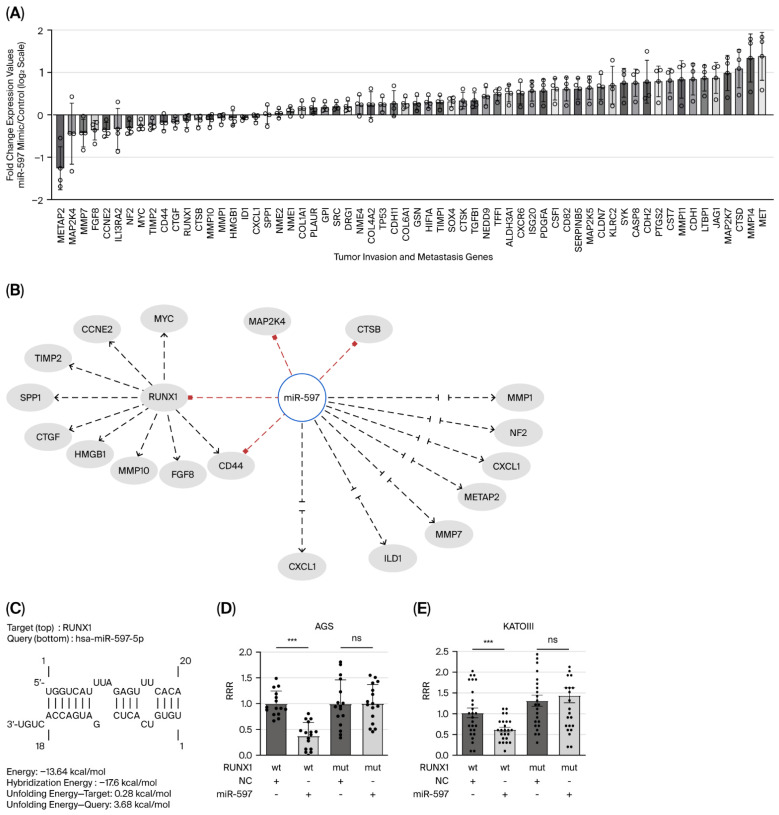
Molecular mechanisms of the tumor-suppressive effects of miR-597. (**A**) PCR array analysis of 62 cell invasion and tumor progression genes after transfection with the miR-597 mimic. Downregulation of 19 genes was observed. (**B**) Visualization with Cytoscape of the regulatory network of miR-597 identified using the ChEA Transcription Factor Targets dataset from the Harmonizome 3.0 database. Four target genes (*RUNX1*, MAP2K, CD44, and CTSB) are directly regulated by miR-597. One of these genes, *RUNX1*, is a hub for nine of the other genes (*SPP1*, *CCNE2*, *FGF8*, *MYC*, *TIMP2*, *CTGF*, *MMP10*, *HMGB1*, and *CD44*). The remaining 7 genes are indirectly regulated by miR-597. (**C**) Predictive binding sequence between *RUNX1* and miR-597 as identified with the IntaRNA program. (**D**,**E**) Relative Response Ratio (RRR) of luciferase activity after co-transfection with miR-597, wild-type *RUNX1* 3-UTR, and mutated *RUNX1* 3-UTR in AGS and KATO III cells (*N* = 3) (*** *p* < 0.001). The *RUNX1* fragment includes the predicted wild-type and mutant target sites of miR-597. Both sites, cloned in a pmirGLO Dual-Luciferase miRNA Target Expression Vector, were co-transfected with the corresponding miR-597 or negative control mimic. Dual-Luciferase^®^ Reporter Assay System was used to detect firefly and Renilla luciferase activities after 24 h of transfection. The wild-type and the mutant sequences of the fragment of the *RUNX1* gene that include the predicted miR-597 target sites were TGGT CATT TAGA GTTT CACA and CTAA ATTT TAGA TTTG CA, respectively. Data are presented as mean ± SD. All readings from the 3 biological replicates are shown. From these readings, outlier results were deleted according to Grubbs’ test.

To elucidate the molecular mechanisms underlying the tumor-suppressive effects of miR-597, we performed a PCR array analysis of 62 cell invasion genes in the AGS cell line after transfection with miR-597 mimic or inhibitor. Nineteen of these genes were downregulated upon transfection with the miR-597 mimic (Figure 3A), while the opposite effect was seen with the miR-597 inhibitor (Figure S2). Using the bioinformatic tool miRPathDB 2.0 [26] to evaluate the interaction between miRNA and mRNAs, we constructed a regulatory network diagram to visualize the interactions between miR-597 and its target genes (Figure 3B). This analysis demonstrated that miR-597 directly regulates 4 of these 19 genes (*RUNX1*, *MAP2K*, *CD44*, and *CTSB*). Analysis of the ChEA Transcription Factor Targets dataset from Harmonizome 3.0 [27] identified that 9 out of the 15 remaining genes (*SPP1*, *CCNE2*, *FGF8*, *MYC*, *TIMP2*, *CTGF*, *MMP10*, *HMGB1*, and *CD44*) depend on *RUNX1* gene regulation (Figure 3B). Notably, high expression of *RUNX1* is associated with poor overall survival when analyzed with the KM plotter tool (Figure S3 [28]). The precise location of the interactions between miR-597 and *RUNX1* was predicted using the IntaRNA program (Figure 3C) [29]. These results reveal that miR-597 suppresses *RUNX1*, a hub for multiple genes involved in cell invasion and tumor progression. To validate the regulatory interplay between miR-597 and *RUNX1*, we employed a luciferase reporter assay. Co-transfection of AGS and KATO III cells with miR-597 and wild-type *RUNX1* 3′-UTR constructs resulted in a significant decrease in the Relative Response Ratio (RRR) of luciferase activity, indicative of a direct interaction between miR-597 and the transcript of *RUNX1*. Mutated *RUNX1* 3-UTR constructs did not exhibit a significant change in the RRR, confirming the specificity of this interaction (Figure 3D,E).

### 2.5. Regulation of miR-597 by Long Non-Coding RNA

**Figure 4:**
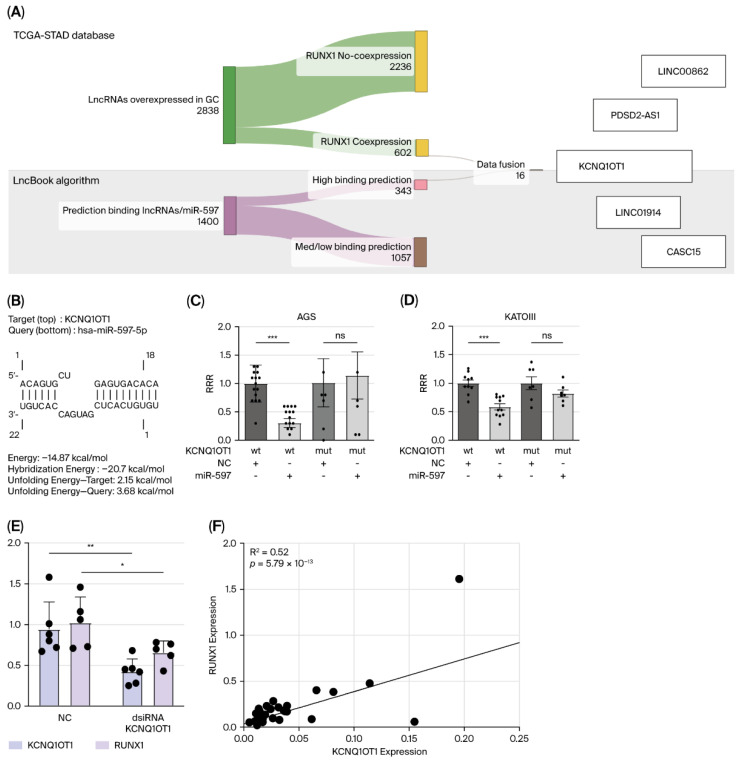
Long non-coding RNA regulates the expression of miR-597. (**A**) Intersection, based on linear regression analysis, between lncRNAs (log fold change > 1 and adjusted *p*-value < 0.05, Benjamini–Hochberg method) overexpressing *RUNX1* (the best target gene for miR-597) from the Cancer Genome Atlas—Stomach Adenocarcinoma (TCGA-STAD) (upper panel) and lncRNAs with a high binding energy level (ΔG) identified with the LncBook tool [30] (lower panel). This data fusion yielded 16 overlapping transcripts. *KCNQ1OT1* is among the top five of these transcripts. (**B**) Predictive binding sequence between *KCNQ1OT1* and miR-597 identified with the IntaRNA program [29]. (**C**,**D**) Relative Response Ratio (RRR) of luciferase activity after co-transfection with miR-597, wild-type *KCNQ1OT1* seed region, and mutated *KCNQ1OT1* seed region in AGS and KATO III cells, respectively (*N* = 3). The wild-type and the mutant sequences of the fragment of the *KCNQ1OT1* gene that include the predicted miR-597 target sites were CGCG TGGA AACA CAGT GCTG AGTG ACAC A and CGCG TGGA AACA CATT CTAC ATCT TG, respectively. Luciferase Reporter assay including the predicted miR-597 target and mutant sites was performed as described in Figure 3C. All readings from the 3 biological replicates are shown. From these readings, outlier results were deleted according to Grubbs’ test. (**E**) Significant reduction in the relative expression of *KCNQ1OT1* and *RUNX1* transcripts following a dicer-substrate siRNA (dsiRNA) pool targeting *KCNQ1OT1* in NCI-N87 gastric cancer cells. NCI-N87, the cell line with the highest *KCNQ1OT1* endogenous expression, was seeded and transfected with a dicer-substrate siRNA pool targeting *KCNQ1OT1* (hs.Ri.*KCNQ1OT1*.13.1, .13.2, .13.3) after 24 h (*N* = 5). (**F**) *RUNX1* and *KCNQ1OT1* co-expression in 69 GC clinical samples (R^2^ = 0.52) (* *p* < 0.05; ** *p* < 0.01 and *** *p* < 0.001). Data are presented as mean ± SD. ns = non-significant.

Having demonstrated the tumor suppressor behavior of miR-597 and the role of its target gene *RUNX1* as a hub for the control of cell invasion and metastasis, we explored the underlying mechanisms of regulation. lncRNAs have emerged as direct modifiers of miR expression due to their sponging capacity; that is, they compete with mRNAs for the same pool of miRs. We analyzed the Cancer Genome Atlas—Stomach Adenocarcinoma (TCGA-STAD) from the dbGaP (ID phs000178) to identify lncRNAs that potentially regulate the availability of miR-597, thereby indirectly targeting *RUNX1*. In total, 2838 lncRNA transcripts exhibiting a significant overexpression (log fold change > 1; adjusted *p*-value < 0.05) in TCGA were selected for linear regression analyses using the best target gene for miR-597, *RUNX1* (Figure 4A, upper panel). This analysis identified 602 lncRNA transcripts. In parallel, an estimate of 1400 lncRNAs that bind miR-597 was obtained using the LncBook tool [30]. These transcripts were filtered based on binding energy levels (ΔG), resulting in a subset of 349 lncRNAs (with high binding prediction). Both subsets were intersected, yielding 16 overlapping transcripts. Among these, the top five lncRNAs with the strongest binding predictions were *KCNQ1OT1*, *PSD2-AS1*, *ZFHX2-AS1*, *ATXN2-AS*, and *SNHG16* (Table S1). *KCNQ1OT1* was chosen for further analysis based on its relevance in GC and other tumors [31,32,33,34]. To identify the specific binding sequence, IntaRNA prediction binding software was employed (Figure 4B). This potential prediction was then validated by co-transfecting miR-597 with the wild-type *KCNQ1OT1* sequence. This analysis resulted in a significant reduction in RRR compared to the controls, whereas mutated *KCNQ1OT1* sequences showed no significant effect (Figure 4C,D). Since miR-597 was predicted and confirmed to bind to both *KCNQ1OT1* and *RUNX1*, we evaluated whether silencing *KCNQ1OT1* affected *RUNX1* expression. *KCNQ1OT1* was knocked down in NCI-N87, the cell line with the highest *KCNQ1OT1* endogenous expression (Figure S4), using a specific dsiRNA, and *RUNX1* transcript levels were significantly reduced (Figure 4E). These findings suggest that *KCNQ1OT1* acts as a miR-597 sponge to modulate *RUNX1* expression. To further confirm the existence of this network, the bivariate expression of *KCNQ1OT1* and *RUNX1* was analyzed in 69 different clinical GC specimens. This analysis revealed a positive correlation between *KCNQ1OT1* expression and *RUNX1* expression (R^2^ = 0.52 and *p* < 0.0001) (Figure 4F). Finally, we ruled out an interaction between *KCNQ1OT1* and *RUNX1* based on LncExpDB 2.0 [35]. Taken together, these results suggest that miR-597 directly targets *RUNX1* to suppress its expression, while *KCNQ1OT1* acts as a miR-sponge to prevent this interaction. Dysregulation of this network may contribute to GC progression, whereby miR-597 is predicted to behave as a tumor suppressor by targeting *RUNX1*, while *KCNQ1OT1* interferes with this suppression to promote oncogenic signaling.

## 3. Discussion

GC is one of the most common malignancies worldwide, with considerable geographic variation [1,2,36]. Understanding the mutational heterogeneity, mostly in genes associated with immune evasion, cell adhesion, and tumor progression in East Asia [3] and HER2 and PD-L1 in Latin America [5] is critical for the development of cost-effective early diagnosis strategies and precise tailored treatments [37,38].

In this study, we provide the first evidence of expression of miR-597 in GC samples, showing that its downregulation correlates with particular clinicopathological characteristics, namely the intestinal subtype according to Lauren classification and advanced stages of disease (stage III and IV). Several miRs have been described in both histopathological subtypes (i.e., intestinal and diffuse), particularly miR-18a, which has been proposed as a prognostic biomarker [39,40]. In the diffuse subtype, miR-199a/b has been associated with advanced stages and has also been proposed as a prognostic biomarker [41]. In the intestinal subtype, upregulation of miR-200b was associated with advanced T stage and worse overall survival [42]. In this subtype, downregulation of miR-1224 has been associated with lymph node metastasis [43].

We investigated the functional role of miR-597 by performing gain- and loss-of-function experiments in AGS and MKN-74 cells, two well-established GC cell lines [44,45], as well as two successfully established PDOs from primary gastric tumors. The miR-597 mimic impaired cell migration and wound healing, while inhibition enhanced them. A reduction in invasiveness and colony formation was also observed after transfection with the miR-597-mimic. Reduction in cell viability was assessed using MTS assays and corroborated using the Trypan blue exclusion assay. These findings were confirmed in a more physiological model, the PDOs. The findings reported here parallel those reported by Zhang [17], who focused on tumor stem cell characteristics in the misidentified cell line SGC-7901 (authenticated as HeLa cells, or a possible hybrid of HeLa with an unknown cell line) [46].

Downregulation of miR-597 has been found in clinical samples from other GI tumors, particularly CRC, HCC, and PAC [18,19,20], but only PAC is associated with poor survival [20]. Gain- and loss-of-function studies showed that miR-597 promoted migration and invasion in CRC cells [20] and reduced cell viability and proliferation in HCC cells [21]. Notably, these features were reversed by the lncRNA GSTM3TV2 through the sponging of miR-597 [21]. In PAC cell lines, miR-597 impaired wound healing ability and colony formation, while reducing invasiveness [19]. Taken together, our results in GC align with these findings, supporting the role of miR-597 as a potential tumor suppressor gene.

The molecular mechanisms of miR-597 for cell invasion and tumor progression were evaluated by screening 62 genes to identify 19 potential candidates. While the majority of these candidates have previously been proposed to promote aggressiveness in GC [47], our regulatory network visualization led us to further narrow the candidate targets down to four genes directly regulated by miR-597: *RUNX1*, *MAP2K*, *CD44*, and *CTSB*. Importantly, the remaining genes (*SPP1*, *CCNE2*, *FGF8*, *MYC*, *TIMP2*, *CTGF*, *MMP10*, *HMGB1*, and *CD44*) depend on *RUNX1* as a hub. This finding shows that miR-597 regulates cell invasion and tumor progression by targeting a single hub gene, *RUNX1*.

While *RUNX1* acts as an oncogene through the activation of the Wnt/beta-catenin and EMT pathways in GI tumors [48], it plays a variety of roles in others [24]. Thus, given that *RUNX1* was identified in GC as a prime target for miR-597, it may be anticipated that the effects of these paired regulators will vary considerably across different tumors. To explore the mechanisms of miR-597 regulation, we evaluated 2838 lncRNA transcripts, which were co-expressed with *RUNX1* in TCGA-STAD. These candidates were intersected with 349 lncRNAs with the highest binding prediction [49], which yielded 16 overlapping transcripts. *KCNQ1OT1* was chosen for further analysis not only because it was one of the top five candidates in this group of overlapping transcripts but also because it represents a promising theragnostic candidate in multiple human tumors [32]. *KCNQ1OT1* spans 91,671 nucleotides and is the longest mono-exonic gene of all annotated transcripts [31]. It is located on chromosome 11p15.5, within a cluster of epigenetically regulated genes, which are transcribed from only one chromosome in a parent-of-origin manner [34]. *KCNQ1OT1* is regulated by a functionally independent imprinting control region located in an intron that is present exclusively in the paternal allele [50]. This gene has been defined as an oncogenic lncRNA in a wide variety of neoplasms in addition to GI tumors [32]. However, controversial findings have been reported regarding the role of *KCNQ1OT1* in GC. One group observed that it promotes GC progression via the miR-556-3p/CLIC1 axis [51]; conversely, Feng et al. [52] have shown that *KCNQ1OT1* inhibits GC progression through the sponging of miR-9 and subsequent upregulation of LMX1A expression. The *KCNQ1OT1* polymorphisms—rs231352, rs7128926, and rs7939976—have been revealed as predictors of susceptibility, shorter recurrence-free survival, and overall survival in GC, respectively [53]. Luciferase reporter assays validated the predicted binding sequence between miR-597 and *KCNQ1OT1*. These results, together with the previous validation of binding between miR-597 and *RUNX1*, suggest the existence of a regulatory network involving these three genes.

**Figure 5:**
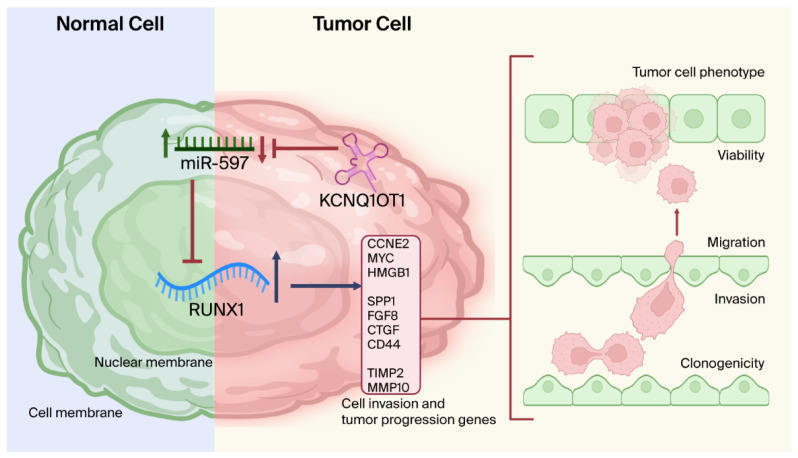
Mechanism of tumor progression regulation mediated by miR-597. This model illustrates the target genes, biological roles, and mechanisms of miR-597 regulation. miR-597 targets *RUNX1*, inducing its endonucleolytic degradation. *RUNX1* acts as a hub for cell invasion and tumor progression genes through other genes (*SPP1*, *CCNE2*, *FGF8*, *MYC*, *TIMP2*, *CTGF*, *MMP10*, *HMGB1*, and *CD44*). *KCNQ1OT1* acts as a negative regulator of miR-597, attenuating the degradation of *RUNX1*. On the right side of the model is a summary of the phenotypes of tumor cells, which are influenced by miR-597, highlighting characteristics such as viability, migration, invasion and clonogenicity.

Further confirmations of this connection include the significantly reduced expression of *RUNX1* upon knockdown of *KCNQ1OT1* in vitro, together with the positive correlation between *KCNQ1OT1* and *RUNX1* expression in clinical samples. Taken together, these findings provide evidence of a recurring framework in which miR-597 suppresses cell invasion and tumor progression via *RUNX1* targeting. This effect is attenuated through the sponging of miR-597 by *KCNQ1OT1*. In this framework, *KCNQ1OT1* and *RUNX1* transcripts compete for miR-597 [54]. As shown in Figure 5, in a normal cell (left panel, light blue zone), high levels of miR-597 promote the endonucleolytic degradation of genes involved in cell invasion and tumor progression, such as *RUNX1*. In a tumor cell (right panel, yellow zone), the downregulation of miR-597 is mediated by the sponging effect of the lncRNA *KCNQ1OT1*. This downregulation leads to the overexpression of *RUNX1*. As a consequence, RUNX1 acts as an oncogene-dysregulating key gene pathway (see insert) associated with cell invasion and tumor progression. Functional assays assessing the phenotype of tumor cells after gain and loss of function of miR-597 confirmed its oncogenic role by promoting clonogenicity, invasion, and migration. The translational value of these findings is the addition of another layer of post-transcriptional regulation that explains the heterogeneity of tumor behavior [55]. The incorporation of these networks built on existing lncRNA–miRNA–mRNA networks might contribute to more robust survival prediction and clinical outcomes [56].

An initial limitation for completing the portrait of miR-597 as a tumor suppressor gene was the use of cell lines, which cannot replicate the complex tumoral environment [57]. To address this issue, we complemented our characterization with PDOs that more closely mimic the biological processes of cell invasion and tumor progression [58]. However, the limited number of PDOs used in this study restrains the generalization of our conclusions. While the AGS cell line is an appropriate primary model for functional assays, it does not fully align with our clinicopathological findings regarding the intestinal subtype according to Lauren classification. Other limitations include the use of different instruments to detect miR-597 expression in clinical samples and cell lines and the lack of additional experiments to confirm a competing endogenous network between *KCNQ1OT1*, miR-597, and *RUNX1*.

In summary, we implemented experimental and translational approaches to characterize miR-597 as a tumor suppressor gene in GC. We analyzed its expression in tumor and adjacent normal tissues using qRT-PCR and evaluated its clinical significance. The gain- and loss-of-function studies performed involved the ectopic expression of miR-597 in cell lines and PDOs, which allowed us to assess its tumor suppressor capacities and its role in promoting oncogenic traits when lost. Bioinformatics algorithms predicted *RUNX1* to be an important transcriptional target of miR-597, an observation that was confirmed in luciferase reporter assays. Through these experiments, the lncRNA *KCNQ1OT1* was also identified as a miR-597 regulator of *RUNX1* endonucleolytic degradation. Our multi-tiered approach enabled the analysis of simultaneous interactions between three families of transcripts (lncRNAs, miRs, and mRNAs) in GC progression; the translational implications of this network cannot be understood by studying each transcript separately.

Further research on the restoration of these interactions will be required to therapeutically reverse the cancer phenotype.

## 4. Materials and Methods

### 4.1. Clinical Samples

Seventy-five decodified fresh frozen tumor samples were obtained from Hispanic-Amerindian patients undergoing total gastrectomy from Hospital Sotero del Rio (HDSR), Metropolitan Region, Chile; Hospital Hernán Henriquez Aravena (HHHA), Araucania Region, Chile; and Tumor and Fluid Bank, Hospital Clinico Universidad de Chile (TFBCH), Metropolitan Region, Chile. In these specimens, paired tumor tissue (T) and normal tissue adjacent to the tumor (NAT) were obtained. Association analysis of the expression of miR-597 with clinicopathological features was performed in these samples. For patient-derived organoid (PDO) establishment, ascites fluid was collected by paracentesis from patients with GC and peritoneal metastasis at the Hospital Clínico Red Salud UC-Christus.

Ethics approval was obtained from the Ethics Committee of the School of Medicine, Universidad De La Frontera, Temuco; the Ethics Committee of Servicio de Salud Metropolitano Sur-Oriente, the Servicio Metropolitano Oriente, Santiago, Chile; and the Institutional Review Board of the School of Medicine of Pontificia Universidad Católica de Chile (ID: 210620005, 7 April 2022). All patients provided written informed consent.

### 4.2. Cell Line and Patient-Derived Organoid Culture Conditions

The expression of miR-597 was evaluated in the AGS cell line because it is both primary-derived and adherent. Three other cell lines (KATO-III, NCI-N87, and MKN74) were chosen for secondary confirmatory assays. The AGS, KATO-III, and NCI-N87 cell lines were obtained from the American Type Culture Collection (ATCC, Manassas, VA, USA) and the MKN74 cell line was kindly donated by Wael El-Rifai. Three of these cell lines (AGS, NCI-N87, and MKN74) were maintained in RPMI-1640 medium (SH30605.01, HyClone, Logan, UT, USA), while KATO-III cells were cultured in IMDM (4 mM L-glutamine, 25 mM HEPES, 25 mM NaHCO_3_); both media were supplemented with 10% heat-inactivated fetal bovine serum (FBS), 10 units/mL penicillin, and 10 mg/mL streptomycin. Cultures were incubated at 37 °C in a humidified atmosphere containing 5% CO_2_ and sub-cultured during the logarithmic growth phase. Patient-derived organoids (PDOs) were generated as previously described by Barker et al. [59] with some minor modifications. The dissociated cells were washed in 1× DPBS and embedded in growth factor-reduced Matrigel (#356231, Corning, NY, USA) and gastric conditioned medium (see supplemental methods). PDOs were passed via mechanical dissociation, and passage was performed weekly with a 1:3 ratio.

### 4.3. RNA Extraction from GC Clinical Samples, Cell Lines, and PDOs

For miRNA isolation samples, the snap-frozen method [60] was employed for tissue disruption and homogenization. The mirVana miRNA Extraction kit (Ambion, Life Technologies, USA) was used to extract miRs from fresh-frozen samples (T and NAT from GC cases) according to the manufacturer’s protocol. For mRNAs and lncRNAs, samples were homogenized using the TissueRuptor II Homogenizer Disruption method according to the manufacturer’s recommendations (Qiagen, Hilden, Germany). RNeasy Mini Kit (Qiagen) RNA extraction was used for mRNA and lncRNA extraction from fresh-frozen clinical tissue samples. For cell lines and PDOs, TRIzol (Thermo Fisher Scientific, Waltham, MA, USA) was used to isolate total RNA from cell lines and PDOs according to the manufacturer’s instructions. In this protocol, we added 20 μg of glycogen prior to isopropyl precipitation. A total of 20 μL of nuclease-free water was used to dissolve the final pellet.

### 4.4. RNA Quantification Using Real-Time qPCR in Clinical Samples, Cell Lines, and PDOs

To evaluate the expression of miR-597, the TaqMan^®^ MicroRNA Reverse Transcription kit (Applied Biosystems, Life Technologies, Carlsbad, CA, USA) was used to reverse-transcribe 10 ng of the extracted mature miRs following the manufacturer’s protocol. For clinical samples, the TaqMan^®^ Universal PCR Master Mix was used with the LightCycler^®^ 480 Instrument (Roche Diagnostics GmbH, Roche Applied Science, Mannheim, Germany) to perform real-time qPCR under the following conditions: initial 2 min UNG activity at 50 °C, 10 min enzyme activation at 95 °C, followed by 45 cycles at 95 °C for 15 s, and 60 °C for 60 s. LightCycler software (release 1.5.0) and the 2^−ΔΔCt^ method [25] were used to determine the cycle threshold (Ct) and calculate the relative gene expression, respectively. For cell lines and PDOs, the same TaqMan^®^ Universal PCR Master Mix was used with the Mic Real-Time PCR Cycler with similar conditions to those described above. Mic-PCR Software (release v2.8.10) and the 2^−ΔΔCt^ method, as mentioned above, were used to determine the Ct and calculate the relative gene expression, respectively. For normalization, a small RNA RNU6B was used as internal control for input. TaqMan^®^ Small Assays for humans were used to obtain predesigned primers and probes (miR-597, assay ID 001551 and RNU6B, assay ID 001093). To detect mRNAs and lncRNAs, we employed the SYBR Green Brilliant II method (Agilent Technologies, Santa Clara, CA, USA) with an AriaMX Real-Time PCR system (Agilent). We performed real-time qPCR with the following conditions: initial denaturation at 95 °C for 10 s; 40 cycles of 95 °C for 30 s, 58 °C for 30 s, and 72 °C for 1 min; followed by a final extension at 72 °C for 10 min and melt curve analysis. AriaMX software (release 2.1.1) was also used to determine the Ct. The 2^−ΔΔCT^ method [25] was used to calculate the relative gene expression. For normalization, GAPDH was used as an internal control for input (Fw GCGA GATC CCTC CAAA ATCA, Rv ATGG TTCA CACC CATG ACGA). Primers for *RUNX1* (Fw TCTT CACA AACC CACC GCAA, Rv TCTG CCG ATGT CTTC GAGGT) were taken from the literature [61]. In-house primers were designed for *KCNQ1OT1* (Fw CTTT GCAG CAAC CTCC TTGT, Rv TGGG GTGA GGGA TCTGAA). All designed primers were evaluated for their efficiency (Table S2). A subset of 69 clinical samples was used to evaluate the co-expression of *KCNQ1OT1* and *RUNX1* via linear regression analysis (see Section 4.10).

### 4.5. Functional Analysis

Transfection experiments in cell lines: 2 × 10^5^ cells were transiently transfected with 30 nM miRNA Mimic and Inhibitor Negative Control#1 (NC, #4464058 mirVana™ Invitrogen™, Waltham, MA, USA), Mimic hsa-miR-597-5p (597m, #4464066 mirVana™ Invitrogen™, Waltham, MA, USA) and inhibitor (597i, #4464084 mirVana™ Invitrogen™, Waltham, MA, USA) using FuGENE^®^ HD Transfection Reagent (Catalog Number: E2311, Promega, Madison, WI, USA), according to the manufacturer’s protocol. Transfection experiments were performed in 6-well plates. Efficiency of transfection was measured based on the expression levels of miR-597 (Figure S2A,B). Transfection experiments in PDOs: 4 × 10^5^ cells were transiently transfected as described above using Lullaby (OZ Biosciences) in Advanced DMEM/F-12 Medium (#12634028; Thermo-Fisher Scientific, USA), according to the manufacturer’s protocol. Briefly, 700 μL of formed mimic complex and 300 μL of cells were seeded into a 6-well ultra-low attachment plate (#3471; Corning, NY, USA) and incubated at 37 °C with 5% CO_2_ for 6 h. Transfected cells were re-counted and plated for clonogenic and viability assays. Transfection efficiency was measured using the expression levels of miR-597 (Figure 3A,D). Following transfections, functional assays were performed. All assays were conducted under standardized conditions to minimize technical variability. For cell migration in transwell assays, Transfected cells were subjected to a migration assay using 8 um pore size polycarbonate membrane transwell chambers in a 24-well plate format (Corning, NY, USA). Then, 4.5 × 10^4^ cells were seeded in serum-free media and allowed to migrate toward 10% FBS for 18 h. Membranes were fixed with methanol and stained with 0.1% crystal violet in 25% methanol/PBS, as described previously [62]. An optical microscope (Zeiss Scope A.1 Axio, 10×, Zeizz, Oberkochen, Germany) was used to count the number of migrating cells. We randomly selected 5 visual fields from each membrane. v1.48r ImageJ^®^ software (NIH, Bethesda, MD, USA) was used to measure migratory abilities, which were expressed as the mean of cells per condition and as a percentage of migratory cells relative to the corresponding NC condition, which was set as 100% for each experiment as previously described [62]. For Wound healing assays, 1 × 10^5^ of transfected cells per well were seeded into 6-well plates. A thin line was scratched with a pipette tip simulating a wound on a monolayer of transfected cells and negative controls. At 3 intervals (0 h, 10 h, and 20 h) after wounding, the migration ability of the cells was evaluated under an inverted microscope. The remaining wounded area was measured with v1.48r ImageJ^®^ software (NIH, USA). Four representative photographs were taken for this purpose. For each time point and condition, the images taken were quantified, and their mean represents one replicate value. The wounded area at 0 h was set as 100% for each replicate, and the remaining wound area at 10 and 20 h was expressed relative to its corresponding 0 h value. For Invasion assays using Matrigel-coated transwells, 7 × 10^4^ of transfected cells in serum-free media were seeded onto filters coated with 20 μg Matrigel (Corning, NY, USA). The chemoattractant (medium containing 10% FBS) was placed in the lower chamber. After 18 h of incubation, a cotton swab was used to remove the cells that did not migrate. The Matrigel-coated membranes were fixed and stained with crystal violet as previously described [62]. Five visual fields from each Matrigel-coated membrane were counted using an optical microscope, and invasive abilities were expressed as described above. For the colony formation assay, 200 transfected cells per well were seeded into a 6-well plate and cultured at 37 °C for 14 days as previously described [63]. Surviving colonies were fixed as described above with methanol for 15–40 min at room temperature, stained with 0.5% crystal violet in 25% methanol/PBS, counted using an optical microscope, and subsequently scanned at high resolution (≥50 cells per colony). Colony formation was expressed as a percentage relative to the corresponding NC condition, which was set as 100% for each experiment. v1.48r ImageJ^®^ software (NIH, USA) was used to analyze surviving colonies, and the results were expressed as the mean of cells per field. To evaluate the clonogenic capacity of miR-597 on PDOs, cells transfected with the miR-597 mimic were resuspended in 2% gastric conditioned medium (see supplemental protocols). Two thousand transfected cells per well were seeded into a 24-well plate and incubated at 37 °C for 14–21 days. Surviving colonies were fixed, stained with Nuclear Fast Red Counterstainer (BSB 0116; Bio SB, Santa Barbara, CA, USA), and counted using an optical microscope (≥50 cells per colony). v1.48r ImageJ^®^ software (NIH, USA) was used to scan images for high-resolution imaging. Colony number was expressed as a percentage relative to the NC condition. Surviving colonies were expressed as the mean number of cells per condition. For the viability assay, 8 × 10^3^ cells per well were seeded in 96-well plates and cultured at 37 °C for 0 h, 24 h, 48 h, and 72 h. At each time point, a 3-(4,5-dimethylthiazol-2-yl)-5-(3-carboxymethoxyphenyl)-2(4-sulfophenyl)-2H-tetrazolium (MTS) reagent (CellTiter 96^®^ AQueous One Solution, Promega, Madison, WI, USA) was added and incubated at 37 °C for 2 h according to the manufacturer’s instructions. A microplate reader (Bio-Rad, Hercules, CA, USA) was used to read the absorbance at 492 nm. For the trypan blue assay, 4 × 10^4^ cells were cultured in 24-well plates and incubated at 37 °C for 12 h, 24 h, and 48 h. Cell suspensions were prepared with 0.4% trypan blue solution, and viable and nonviable cells were counted in a Neubauer chamber. Cell viability was expressed as the percentage of viable cells relative to the total number of counted cells.

### 4.6. PCR Array and Network Analysis of Cell Invasion and Tumor Progression Genes

To identify the targets of miR-597 associated with cell invasion and tumor progression, we performed a PCR array (Pathway PCR array, Real Time Primers, Elkins Park, PA, USA) on AGS cells transfected with the miR-597 mimic or inhibitor. This targeted array contained 88 genes and 8 housekeeping gene primer sets in a 96-well microplate. Three reference genes (*RPL13A*, *ACTB*, and *PPIA*) were chosen using geNorm v3.5 software for normalization [60]. Relative gene expression was calculated using the Livak method [25], followed by log2 fold change analysis relative to the negative control. Genes showing lower or higher expression than the negative were considered downregulated or upregulated, respectively. Dysregulated genes from the PCR array, after overexpression with mimic hsa-miR-597-5p, are as described above. All 19 genes downregulated upon miR-597 mimic overexpression were loaded into miRPathDB 2.0 [26]. The Harmonizome 3.0—ChEA Transcription Factor Targets dataset was used to identify genes that depend on *RUNX1* gene regulation [27], and visualization of these regulatory networks was constructed with Cytoscape 3 [64]. The IntaRNA program v3.4.1 [29] was employed to predict binding sites for interaction between miR-597 and the selected mRNA, *RUNX1*.

### 4.7. Luciferase Reporter

In brief, a fragment of *RUNX1* including the in silico predicted miR target sites (TGGT CATT TAGA GTTT CACA) or the mutant (CTAA ATTT TAGA TTTG CA) target site was cloned downstream of the Firefly luciferase gene in a pmirGLO Dual-Luciferase miRNA Target Expression Vector (Cat. #E1330, Promega). Another fragment of KCNQ1OT, including the in silico predicted miR target sites (CGCG TGGA AACA CAGT GCTG AGTG ACAC A) or the mutant (CGCG TGGA AACA CATT CTAC ATCT TG) target site, was also cloned as previously described [65]. Next, 2 × 10^4^ AGS or KATO III cells were placed in 96-well plates and co-transfected with luciferase plasmids and the corresponding miR-597 or negative control mimic. After 24 h of transfection, Firefly and Renilla luciferase activities were detected using the Dual-Luciferase^®^ Reporter Assay System (Cat. #E1910, Promega). Firefly luminescence was normalized to Renilla luciferase activity, which is constitutively expressed by the pmirGLO vector as an internal transfection control. The relative reporter ratio (RRR) was calculated as RRR = [(Firefly/Renilla)experimental]/[(Firefly/Renilla)control], where “experimental” and “control” refer to cells co-transfected with the WT or MUT construct plus the miR-597 mimic or a negative control mimic, respectively.

### 4.8. Knockdown of the Long Non-Coding KCNQ1OT1

NCI-N87 cells were seeded at a density of 4 × 10^5^ cells per well into 6-well plates and incubated for 24 h. Cells were then transfected with 10 nM of a Dicer-substrate siRNA pool targeting *KCNQ1OT1* (TriFECTa^®^ Kit; Integrated DNA Technologies; IDs: hs.Ri.*KCNQ1OT1*.13.1, .13.2, .13.3) using Lullaby transfection reagent (OZ Biosciences, Marseille, France), following the manufacturer’s instructions.

### 4.9. In Silico Analysis for Long Non-Coding RNAs

LncRNAs from the Cancer Genome Atlas—Stomach Adenocarcinoma (TCGA-STAD) dbGaP (ID phs000178) were analyzed. LncRNA transcripts exhibiting a significant overexpression (log fold change > 1; adjusted *p*-value < 0.05, Benjamini–Hochberg method) were selected for linear regression analyses with *RUNX1*, the best target gene for miR-597. The LncBook tool [30] was employed to predict lncRNAs that bind miR-597 based on binding energy levels (ΔG). Both subsets were intersected, yielding the top overlapping transcripts, among which *KCNQ1OT1* was selected for validation analysis. IntaRNA prediction software was employed to identify the specific binding sequences [29]. LncExpDB 2.0 [35] was used to evaluate potential interactions between *KCNQ1OT1* and *RUNX1*.

### 4.10. Statistical Analysis

Statistical analyses were performed using GraphPad Prism 10.6.1 (GraphPad Software Inc., San Diego, CA, USA) and R studio [66]. All statistics were expressed as the mean ± standard deviation (SD) of 3 independent experiments. To compare two groups, we employed a *t*-test. For comparison of multiple groups, the ANOVA test was performed with the corresponding post-test. *p* < 0.05 was considered statistically significant (* *p* < 0.05; ** *p* < 0.01 and *** *p* < 0.001). For the co-expression analysis, we employed the linear regression method [66]. Analysis of the non-significant variables in Table 1 (Sex, Age, and Location) was performed, and the results are shown in Table S3.

## Figures and Tables

**Table 1:** Clinicopathological correlations of miR-597 (*N* = 75 GC samples).

Variable	Category	Frequency	miR-597 Exp * Mean (SD)	*p*-Value	Missing Data
Sex				0.072	0
	male	46 (61.3%)	0.021 (0.022)		
	female	29 (38.7%)	0.013 (0.016)		
Age				0.364	3 (4.0%)
	<50	6 (8.0%)	0.017 (0.012)		
	50–70	43 (57.3%)	0.021 (0.024)		
	>70	23 (30.7%)	0.014 (0.012)		
Location				0.57	10 (13.3%)
	proximal	17 (13.3%)	0.022 (0.027)		
	body	27 (36.0%)	0.014 (0.013)		
	body and distal	8 (10.7%)	0.018 (0.025)		
	distal	13 (17.3%)	0.015 (0.014)		
Histology				0.002	1 (1.3%)
	intestinal	47 (62.7%)	0.012 (0.013)		
	diffuse	21 (28.0%)	0.030 (0.028)		
	mixed	6 (8.0%)	0.019 (0.013)		
Differentiation				0.044	3 (4.0%)
	well	11 (14.7%)	0.024 (0.020)		
	moderately	27 (36.0%)	0.010 (0.010)		
	poorly	34 (45.3%)	0.022 (0.024)		
Stage				0.048	2 (2.7%)
	I–II	31 (41.3%)	0.024 (0.024)		
	III–IV	43 (56.0%)	0.014 (0.017)		

* Mean and standard deviation (SD) were analyzed using the 2^−ΔCT^ method.

## Data Availability

The data presented in this study are openly available I https://figshare.com/account/articles/31955859 (accessed on 1 April 2026).

## Supplementary Materials

The following supporting information can be downloaded at https://www.mdpi.com/article/10.3390/ijms27125368/s1.

## Abbreviations

The following abbreviations are used in this manuscript:

GC	Gastric Cancer
miR/microRNA	MicroRNA
lncRNA	Long non-coding RNA
mRNA	Messenger RNA
nt	Nucleotides
PDOs	Patient-derived organoids
NAT	Normal tissue adjacent to the tumor
EMT	Epithelial–mesenchymal transition
CRC	Colorectal cancer
HCC	Hepatocellular carcinoma
PAC	Pancreatic cancer
3′-UTR	3′ Untranslated Region
HER2	Human epidermal growth factor receptor 2
PD-L1	Programmed death-ligand 1
qRT-PCR/RT-PCR	Quantitative Real-Time Polymerase Chain Reaction
NC	Negative Control
RRR	Relative Response Ratio
MTS	3-(4,5-dimethylthiazol-2-yl)-5-(3-carboxymethoxyphenyl)-2(4-sulfophenyl)-2H-tetrazolium
dsiRNA	Dicer-substrate siRNA
SD	Standard Deviation
ANOVA	Analysis of variance
TCGA-STAD	The Cancer Genome Atlas—Stomach Adenocarcinoma
ACCDiS	Advanced Center for Chronic Diseases
ICBM	Institute of Biomedical Sciences
CEMC	Center for Studies on Exercise, Metabolism and Cancer
HDSR	Hospital Dr. Sotero del Río
HHHA	Hospital Hernán Henríquez Aravena
TFBCH	Tumor and Fluid Bank, Hospital Clínico Universidad de Chile
dbGaP	Database of Genotypes and Phenotypes
*KCNQ1OT1*	KCNQ1 opposite strand/antisense transcript 1
*RUNX1*	Runt-related transcription factor 1
