# Supplementary material for: MicroRNA-597 Suppresses Gastric Cancer Invasion and Progression via RUNX1 Targeting, an Effect Attenuated by the Long Non-Coding RNA KCNQ1OT1
Source: Int J Mol Sci. 2026 Jun 14;27(12):5368. doi: 10.3390/ijms27125368 (PMC13299258; doi:10.3390/ijms27125368)
Supplement: Supplementary file 1 [file ijms-27-05368-s001.zip › supplementary figure legends.docx.pdf]

SUPPLEMENTAL FIGURE LEGENDS

Supplemental Figure 1: A. Relative expression of miR-597 in GC cell lines. B. Relative expression of miR-597 in AGS cell line after transfection with miR-597 mimics and miR-597 inhibitor. C. Cell viability after transfection with miR-597 mimics measured by trypan blue assay in AGS cell line: quantification of cell viability at 12 h, 24 h, and 48 h post-transfection. D. Relative expression of miR-597 after transfection in the MKN74 cell line with miR-597 mimics (N=3). (** $p<0.01$, *** $p<0.001$).

Supplemental Figure 2: PCR array of tumor invasion and metastasis genes after transfection with the miR-597 inhibitor (N=3).

Supplemental Figure 3: Survival curve according to RUNX1 expression in intestinal GC cases (N=179 cases) from the KM plotter data. HR: hazard ratio.

Supplemental Figure 4: Relative expression of KCNQ1OT1 in GC cell lines. (*** $p<0.001$).
