# Supplementary material for: MicroRNA-597 Suppresses Gastric Cancer Invasion and Progression via RUNX1 Targeting, an Effect Attenuated by the Long Non-Coding RNA KCNQ1OT1
Source: Int J Mol Sci. 2026 Jun 14;27(12):5368. doi: 10.3390/ijms27125368 (PMC13299258; doi:10.3390/ijms27125368)
Supplement: Supplementary file 1 [file ijms-27-05368-s001.zip › supplementary Methods_2605130853.docx.pdf]

Supplemental Methods

Gastric conditional medium for patients derived organoids culture

- L-WRN fibroblast conditioned medium, 0.5 μmol/L A83-01 (#SML0788; Sigma-Aldrich)
- 100 ng/mL, FGF10 (#100-26, PeproTech)
- 10 nmol/L Gastrin I (#G9145; Sigma-Aldrich)
- 1 mmol/L N-acetyl-L-cysteine (#A9165; Sigma-Aldrich)
- 10 mmol/L nicotinamide (#N0636; Sigma-Aldrich)
- 1× B27 supplement (#17504-044; Gibco)
- 1× N2 supplement (#175020-048; Gibco)
- 1 mg/mL Primocin (#ant-pm-1; InvivoGen)
- 10.5 μmol/L Y-27632 (#Y0503, Sigma-Aldrich), 0.5 μmol/L Prostaglandin E2 (#P0409-1ug; Sigma-Aldrich)
- 7.5 μg/ml Insulin (#I9278-5ML; Sigma-Aldrich), 10 μmol/L SB202190 (#S7067; Sigma-Aldrich) and 10μg/mL Transferrin (#T8158; Sigma-Aldrich).
