# Supplementary material for: MicroRNA-597 Suppresses Gastric Cancer Invasion and Progression via RUNX1 Targeting, an Effect Attenuated by the Long Non-Coding RNA KCNQ1OT1
Source: Int J Mol Sci. 2026 Jun 14;27(12):5368. doi: 10.3390/ijms27125368 (PMC13299258; doi:10.3390/ijms27125368)
Supplement: Supplementary file 1 [file ijms-27-05368-s001.zip › supplementary Table 1 Intersection TCGA and lncBook.pdf]

sTable 1. Intersection of the top 16 lncRNAs based on the high binding energy levels (ΔG) (lncBook) and coexpression with RUNX1 (TCGA-STAD)

lncRNA symbol	lncRNA ID	coef coexpression	std error	P value	R squared	FDR	miRNA binding	start bindig site	end binding site	energy	software evidence
PSD2-AS1	ENSG00000249131	1,5842	0,4131	0,0001	0,0319	0,0019	miR-597-5p	113	134	-28,19	miRanda,TargetScan
KCNQ1OT1	ENSG00000269821	0,2191	0,0786	0,0056	0,0171	0,0328	miR-597-5p	4397	4418	-26,70	miRanda,TargetScan
CASC15	ENSG00000272168	1,2538	0,1921	0,0000	0,0872	0,0000	miR-597-5p	35	56	-26,19	miRanda,RNAhybrid
LINC00862	ENSG00000203721	0,6709	0,2253	0,0031	0,0195	0,0208	miR-597-5p	194	214	-25,94	miRanda,TargetScan
LINC01914	ENSG00000234362	0,4860	0,0822	0,0000	0,0727	0,0000	miR-597-5p	6336	6356	-23,29	miRanda,TargetScan
CYP4A22-AS1	ENSG00000225506	0,3028	0,0971	0,0019	0,0213	0,0146	miR-597-5p	19	41	-23,19	miRanda,TargetScan
LNCAROD	ENSG00000231131	0,3962	0,1055	0,0002	0,0307	0,0024	miR-597-5p	1593	1614	-22,97	miRanda,TargetScan
LINC02561	ENSG00000224034	0,5585	0,1967	0,0047	0,0177	0,0290	miR-597-5p	297	317	-22,38	miRanda,TargetScan
CCDC144NL-AS1	ENSG00000233098	0,2324	0,0693	0,0009	0,0246	0,0075	miR-597-5p	246	268	-22,06	miRanda,TargetScan
PELATON	ENSG00000224397	0,5344	0,0662	0,0000	0,1276	0,0000	miR-597-5p	271	291	-22,04	miRanda,TargetScan
DDX11-AS1	ENSG00000245614	0,5239	0,1823	0,0042	0,0182	0,0267	miR-597-5p	3205	3225	-21,89	miRanda,TargetScan
RHPN1-AS1	ENSG00000254389	0,2815	0,0654	0,0000	0,0398	0,0004	miR-597-5p	5645	5664	-21,59	miRanda,TargetScan
YEATS2-AS1	ENSG00000233885	1,2789	0,2125	0,0000	0,0751	0,0000	miR-597-5p	36	59	-21,59	miRanda,TargetScan
LINC01517	ENSG00000232624	0,3106	0,1017	0,0024	0,0205	0,0172	miR-597-5p	819	840	-21,32	miRanda,TargetScan
LINC01094	ENSG00000251442	0,8897	0,0963	0,0000	0,1605	0,0000	miR-597-5p	2584	2609	-21,11	miRanda,TargetScan
PRECSIT	ENSG00000255874	0,3140	0,0690	0,0000	0,0443	0,0001	miR-597-5p	799	818	-20,53	miRanda,TargetScan
