# Supplementary material for: MicroRNA-597 Suppresses Gastric Cancer Invasion and Progression via RUNX1 Targeting, an Effect Attenuated by the Long Non-Coding RNA KCNQ1OT1
Source: Int J Mol Sci. 2026 Jun 14;27(12):5368. doi: 10.3390/ijms27125368 (PMC13299258; doi:10.3390/ijms27125368)
Supplement: Supplementary file 1 [file ijms-27-05368-s001.zip › Supplementary Table 2 Primers efficiency.pdf]

Supplementary Table 2 Primers efficiency

GAPDH efficiency curve

sample	dilution	Cq1 GAPDH	Cq2 GAPDH	mean			
1	1	19.56	19.65	19.61			
2	0.1	20.73	20.9	20.82			
3	0.01	24.21	24.21	24.21			
4	0.001	28.12	27.65	27.89			
5	0.0001	32.19	32.01	32.10			
6	0.00001	39.77	35.16	37.47			
7	0.000001	No Cq	No Cq				
	concentration	Log10 C	Cq mean	R2	slope	efficiency	%
1	1000000	6	19.61	0.98	-3.29	2.01	101.16
2	100000	5	20.82				
3	10000	4	24.21				
4	1000	3	27.89				
5	100	2	32.10				
6	10	1	35.16				

RUNX1 efficiency curve							
sample	dilution	Cq1 RUNX1	Cq2 RUNX1	Cq3 RUNX1	mean		
1	1	10.02	10.17	9.82	10.00		
2	0.1	13.56	13.26	13.32	13.38		
3	0.01	16.18	16.49	16.39	16.35		
4	0.001	19.68	19.59	19.43	19.57		
5	0.0001	22.99	22.84	23.04	22.96		
6	0.00001	26.57	25.96	26.25	26.26		
7	0.000001	27.93	27.6	27.89	27.81		
	concentration	log10 C	Cq mean	R2	slope	efficincy	%
1	1000000	6	10.00	0.99	-3.24	2.04	103.76
2	100000	5	13.38				
3	10000	4	16.35				
4	1000	3	19.57				
5	100	2	22.96				
6	10	1	26.26				
7	1	0					

KCNQ1OT1 efficiency curve							
sample	dilution	Cq1	Cq2	mean			
1	1	9.75	9.46	9.61			
2	0.1	12.90	12.60	12.75			
3	0.01	15.69	15.67	15.68			
4	0.001	18.93	19.05	18.99			
5	0.0001	22.28	22.18	22.23			
6	0.00001	25.69	25.51	25.60			
7	0.0000001	28.66	28.53	28.60			
	concentration	Log10 C	Cq mean	R2	slope	efficiency	%
1	1000000	6	9.61	0.99	-3.19	2.06	105.72
2	100000	5	12.75				
3	10000	4	15.68				
4	1000	3	18.99				
5	100	2	22.23				
6	10	1	25.60				
7	1	0					
