# Supplementary material for: MicroRNA-597 Suppresses Gastric Cancer Invasion and Progression via RUNX1 Targeting, an Effect Attenuated by the Long Non-Coding RNA KCNQ1OT1
Source: Int J Mol Sci. 2026 Jun 14;27(12):5368. doi: 10.3390/ijms27125368 (PMC13299258; doi:10.3390/ijms27125368)
Supplement: Supplementary file 1 [file ijms-27-05368-s001.zip › supplementary Table 3 Power table.pdf]

Supplemental Table 2. Power table

Variable	Category	Frequency	miR-597 exp mean (sd)	Effect size	Power post-hoc	Sample size by 80% power
Sex				0,4	38%	200
	male	46 (61.3%)	0.021 (0.022)			
	female	29 (38.7%)	0.013 (0.016)			
Age				0,19	27%	273
	<50	6 (8.0%)	0.017 (0.012)			
	50-70	43 (57.3%)	0.021 (0.024)			
	>70	23 (30.7%)	0.014 (0.012)			
Location				0,16	16%	436
	proximal	17 (13.3%)	0.022 (0.027)			
	body	27 (36.0%)	0.014 (0.013)			
	body and distal	8 (10.7%)	0.018 (0.025)			
	distal	13 (17.3 %)	0.015 (0.014)			
