# Supplementary figures and images for: MicroRNA-597 Suppresses Gastric Cancer Invasion and Progression via RUNX1 Targeting, an Effect Attenuated by the Long Non-Coding RNA KCNQ1OT1
Source: Int J Mol Sci. 2026 Jun 14;27(12):5368. doi: 10.3390/ijms27125368 (PMC13299258; doi:10.3390/ijms27125368)

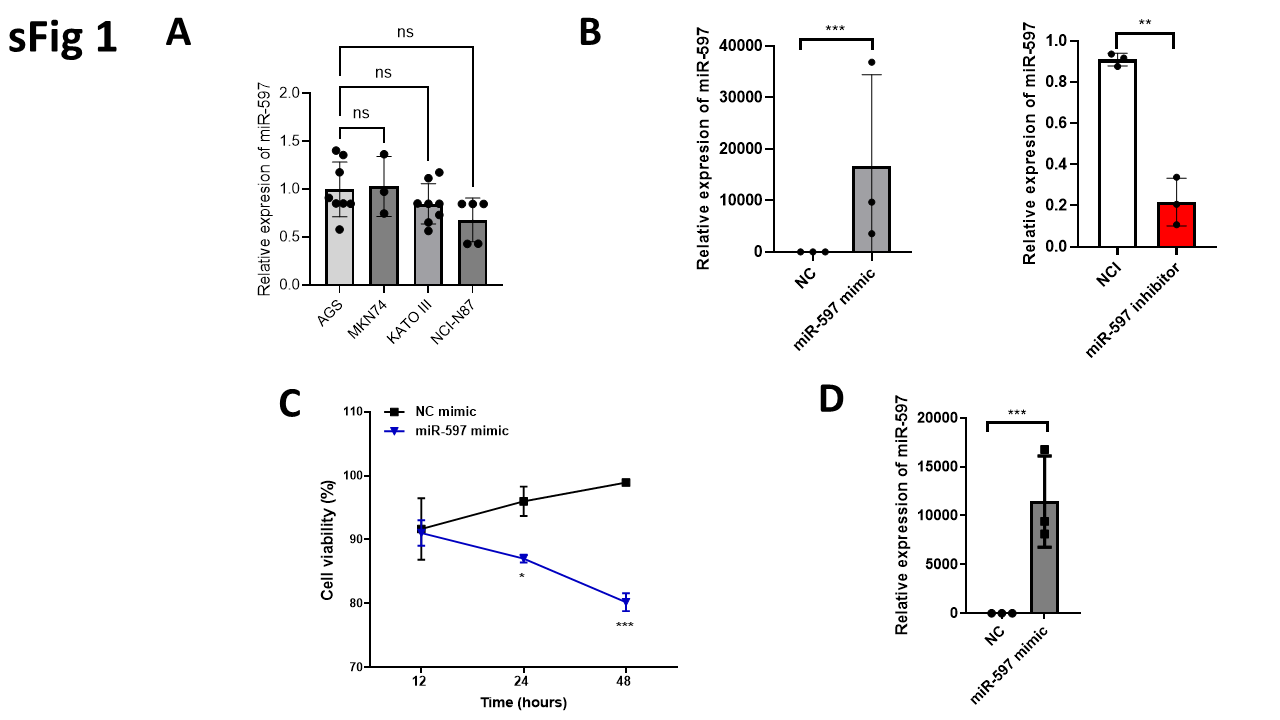

Supplement: Supplementary file 1 [file ijms-27-05368-s001.zip › sFig 1.TIF]

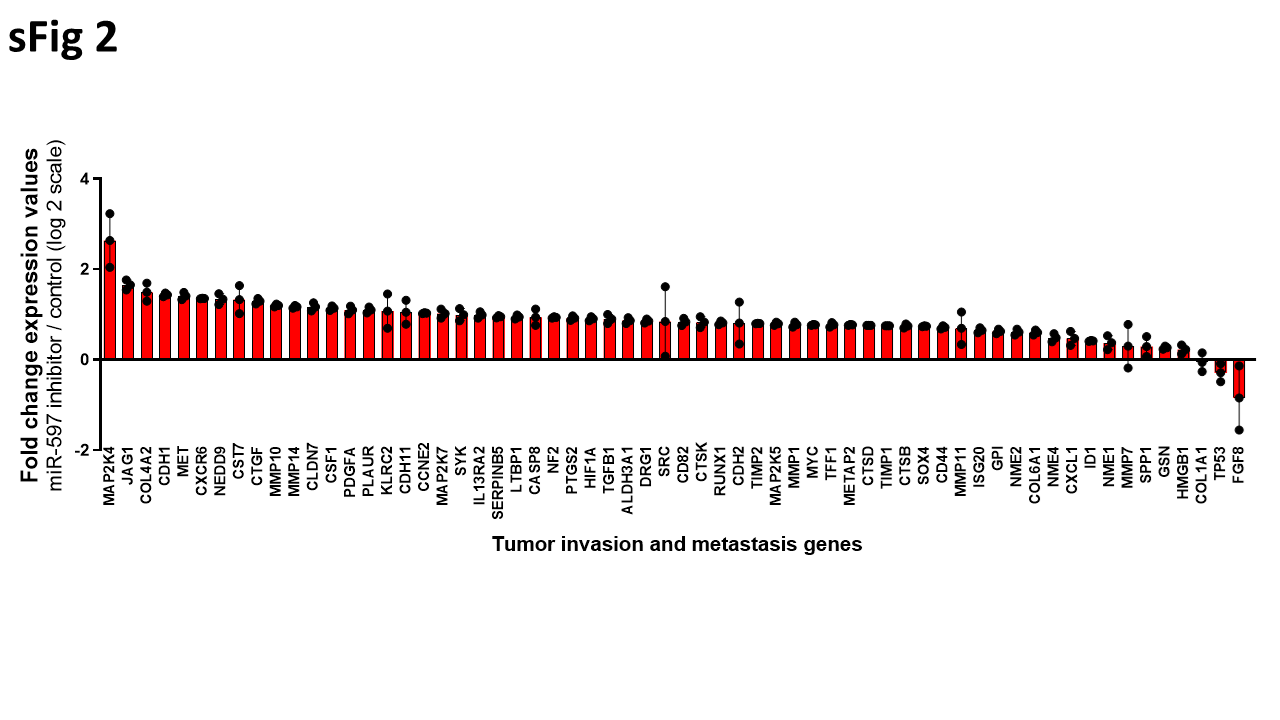

Supplement: Supplementary file 1 [file ijms-27-05368-s001.zip › sFig 2.TIF]

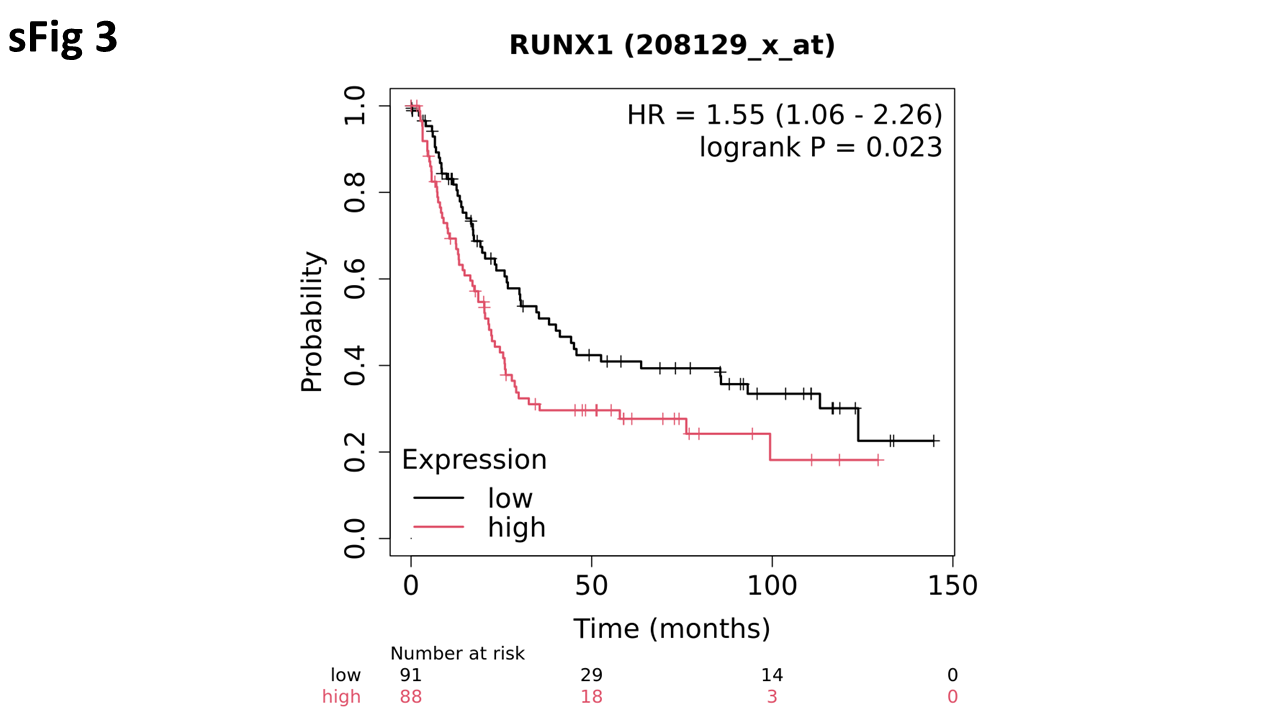

Supplement: Supplementary file 1 [file ijms-27-05368-s001.zip › sFig 3.TIF]

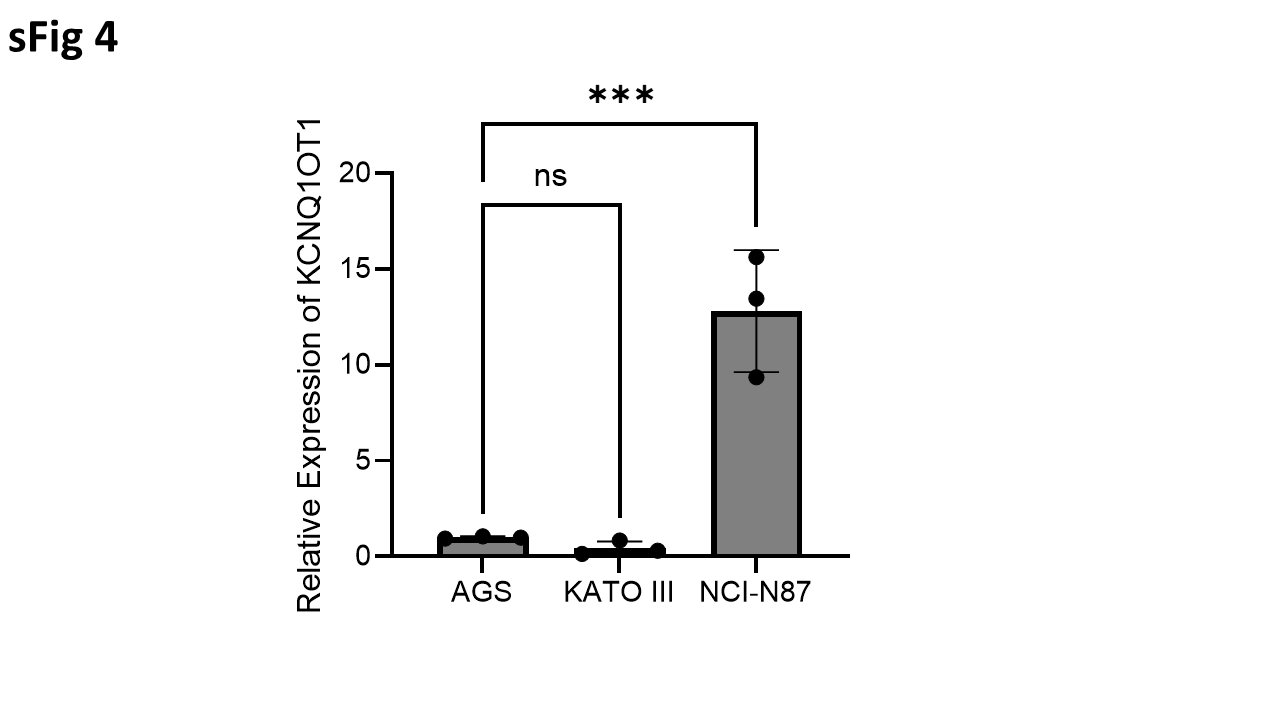

Supplement: Supplementary file 1 [file ijms-27-05368-s001.zip › sFig 4.TIF]
